# Supply Chain and Delivery of Antimicrobial Drugs in Smallholder Livestock Production Systems in Uganda

**DOI:** 10.3389/fvets.2021.611076

**Published:** 2021-09-08

**Authors:** Michel Mainack Dione, Winfred Christine Amia, Francis Ejobi, Emily Awuor Ouma, Barbara Wieland

**Affiliations:** ^1^Animal and Human Health Program, International Livestock Research Institute, Dakar, Senegal; ^2^Animal and Human Health Program, International Livestock Research Institute, Kampala, Uganda; ^3^College of Veterinary Medicine, Animal Resources and Biosecurity, Makerere University, Kampala, Uganda; ^4^Animal and Human Health Program, International Livestock Research Institute, Addis Ababa, Ethiopia; ^5^Institute of Virology and Immunology, Mittelhaeusern, Switzerland

**Keywords:** antibiotic, antimicrobial resistance, livestock, veterinary drug supply chain, Uganda

## Abstract

This study assessed the veterinary drug supply chain in Uganda, the constraints faced by the actors, and how the challenges influence the use of antimicrobial (AMs) by livestock farmers. We carried out stakeholder consultation workshops, key informant interviews and a knowledge, practices, and awareness survey with actors of the veterinary drug supply chain. We also profiled drugs stored in 23 urban and peri-urban drug shops in Lira and Mukono districts to record the commonly sold drugs. The veterinary drug supply chain is made of several actors including wholesalers, retailers, Animal Health Service Providers (AHSP) and farmers. Nearly ninety per cent of drug retailers and veterinary practitioners did not receive specialized training in veterinary medicine, and most of veterinary practitioners have been in the drug business market for more than 10 years. Antibiotics and anti-helminthics were the most stocked drugs by retailers, with antibiotics ranking highest in terms of contribution to annual financial profits, accounting for 33%. The choice of a drug by veterinary practitioners was mainly informed by past success with efficacy of the drug, and financial capacity of the client (the farmer) to meet the treatment cost. Many veterinary practitioners were not conversant with veterinary drug policies of the country, with Mukono having a higher number (72%) compared to Lira (37%). Veterinary practitioners from Lira district compared to Mukono and those mainly serving small scale farmers relative to large scale smallholders were more knowledgeable about antibiotics and AMR. Several supply chain constraints were identified as potential drivers of misuse of antibiotics that could contribute to AMR. These included low level of education of supply chain actors, particularly drug retailers, poor handling of drugs at purchase and administration practices, low enforcement of policies and regulations, and lack of awareness of stakeholders about policies that regulate drug use. Thus, future interventions to reduce misuse of AM drugs in livestock production systems in Uganda such as capacity building, should also target veterinary input suppliers, and deliberately involve a strong policy advocacy component.

## Introduction

While access to quality drugs by livestock producers remains a challenge, there is also misuse of drugs that are easily accessible. The voluntary and involuntary misuse of veterinary drugs such as antimicrobials (AM) in food-producing animals has the potential to generate residues in animal derived products (meat, milk, eggs and honey) and poses a health hazard to the consumer (1). For example, Dar et al. (2) reported that there is a high level of farmer self-prescription, with around a third of countries allowing over-the-counter sales of drugs. This situation is not only an indicator of poor-quality animal health services and animal husbandry practices but may also result in an increase in AMR risks in both animals and humans. While high income countries have improved structures to better monitor quality and quantity of veterinary drugs in the market, as well as testing AM residues in animal source foods, most low- and middle-income countries such as Uganda face challenges to put in place adequate systems to monitor veterinary drugs use in the livestock sector. Since the structural adjustment programs in the 1980s, in Uganda, like in many other sub-Saharian countries, the private sector has contributed immensely to the delivery of animal health services, and government's role has become largely regulatory (3). However, implementation of regulations and policies that govern the delivery of veterinary drugs is a challenge due to lack of resources and commitment of the government (4). Veterinary input suppliers such as drug wholesalers, retailers and veterinary practitioners are important actors in the drug supply chain, since they play an important role in ensuring quality of products to livestock farmers (5, 6). However, few studies have addressed veterinary drugs supply chain issues, especially in relation to AMR. The present study is a first step toward a comprehensive assessment of the use of AMs in smallholder livestock systems in Uganda. Its objectives are to understand the veterinary drug supply chain, constraints faced by actors, assess knowledge, practices, and awareness of veterinary drug suppliers on drug use and management practices, document policy gaps and how they influence the use of AMs. The study was implemented using three approaches: (1) qualitative assessment and description of the veterinary drug supply chains and its challenges; (2) profiling of veterinary drugs stocked by retailers and description of sales practices and (3) a KAP survey with veterinary practitioners about AMR and their perception of policies that govern the sale of veterinary drugs.

## Materials and Methods

### Study Sites

The study was conducted in Kampala, Mukono, and Lira districts in Uganda. Kampala is the capital city of Uganda from where major drug wholesalers carry out their business. Mukono district is in central Uganda located 40 km from Kampala, with a population of 596,804 people, of which 59% are involved in agriculture (7). Because of the proximity to Kampala, livestock farmers are assumed to have better access to veterinary drugs and other animal health inputs. Lira District is in Northern Uganda, about 300 km from Kampala, had an estimated human population of 377, 800 in 2010. The economy of the district is mainly based on agriculture, with 81% of the population engaged in subsistence farming with cattle being the main source of wealth, and bulls and oxen being a major source of traction, particularly for plowing (8). Piggery has increasingly become an important enterprise with 40% of sub-counties having piggery as a priority enterprise (9). The two districts were purposively chosen for their contrasting geographic situations to enable comparison in relation to implications of the locations on potential quality of veterinary inputs and their access by farmers.

### Data Collection

#### Qualitative Assessment of the Veterinary Drug Supply Chains

##### Consultation With Stakeholders of the Veterinary Drug Supply Chains

Two workshops were held with stakeholders of the veterinary drug supply chains in Mukono district (May 2016) and Kampala (December 2017). These two areas were targeted because of their high density of veterinary drugs shops. The workshops were organized and facilitated by the researchers. Workshop reports were developed at the end of each session. The discussions during the workshops covered challenges faced by actors, and recommendations to improve drug supply chains.

In Mukono, the workshop brought together private sector such as drug stockists, government [District Veterinary Officer (DVO) and drug inspector] and regulators [National Drug Authority (NDA)]. The stakeholders were also sensitized on the importance of application of best practices involving drug use, handling, and storage.

In Kampala, participants in the workshop included public and private veterinary services, drug retailers and the NDA and researchers (Table 1).

**Table 1:** Profile of participants to different activities.

**Activity**	**Location**	**Target stakeholder**	**Target information**	**Number of participants**
**Qualitative assessment and description of the veterinary drug supply chains**				
Stakeholder consultation workshop	Mukono	Private veterinarian (16), Drug stockist (7), Researchers (2), National Drug Authority (1), Senior Veterinary Inspector (1), District Veterinary Officer (1), District Animal Health Production Officer (1)	Document constraints in the drug supply chains at district level	29
	Kampala	District Veterinary Officer (1); Drug stockist (2); National Drug Authority officer (1); Researcher (1).	Document constraints in the drug supply chains at district level	5
Key Informant Interview	Kampala^*^	Distributor (primary wholesaler)	Understand the drug supply chain	2
**Veterinary drug profiling and drug sale practices**				
Drug profiling and informal interview with drugs retailers and field observations^**^	Lira	Drug retailers	Types of veterinary drugs sold and document sale practices	10
	Mukono	Drug retailers		13
**Quantitative survey on knowledge and awareness about AMR with veterinary practitioners**				
Survey on knowledge and awareness of AMR and related policies	Lira	Veterinary practitioners	knowledge, practices, and awareness on drug use an awareness about policies and regulations	32
	Mukono	Veterinary practitioners		68

* *All large-scale veterinary drug distributors are in Kampala*.

** *Observations happened during the drug profiling in the same drug shops*.

##### Key Informant Interviews With Large-Scale Drug Distributors (or Primary Wholesalers)

Independent in-depth face-to-face informal interviews were carried out with executives of the two major veterinary drug companies in the country at their headquarters in Kampala in November 2017. The discussions focused on description of the veterinary drug supply chains, as well as the regulatory framework for drugs distribution (Table 1).

#### Veterinary Drug Profiling and Description of Drug Sale Practices

##### Profiling of Veterinary Drugs Sold in the Market

The veterinary drug profiling survey was carried out by researchers in Lira and Mukono districts in August and September 2018, respectively. A list of registered veterinary drug retail shops was obtained from the DVO of the respective districts. Ten drug retailers in Lira and 13 in Mukono districts were included in the study. Most drug shops were in urban and peri-urban areas. The drug profiling exercise included recording of all drugs that were stocked in these shops at the time of the visits. The visit lasted an average of 4 h for each of the drug shops. Each drug was listed independently by trade name, and other data that were captured using photographic images included the active compound of the drug and indication for diseases which drugs identified could be used to treat. The qualification of the retailer and the number of years a specific drug has been on the market were recorded (Table 1).

##### Informal Interview With Drug Retailers and Field Observations

This activity was carried out purely *ad-hoc*. During the visits to the drug shops, interactions between the buyers and the drug stockists were observed and recorded. The researchers also engaged in informal discussions whenever necessary with the drug stockists to better understand their management practices and those of livestock keepers (Table 1).

### Survey With Veterinary Practitioners on Knowledge, Practices, and Awareness About AMR and Perception of Related Policies

A list of all registered veterinary practitioners in urban and peri-urban areas was provided by the DVO of the respective districts. All registered veterinary practitioners were interviewed in both districts (Lira, 32 and Mukono, 68). Data on knowledge, practices of drug use and awareness of AMR were collected through a structured questionnaire. The questionnaire was digitalized through ODK (Open Data Kit) installed on tablets and uploaded to a server. The questionnaire was pre-tested by the researchers and refined before being administered by trained extension agents who operate in the study areas (Table 1).

### Data Management and Analysis

#### Qualitative Analysis

The qualitative data was collected by researchers in notebooks during workshops, interviews, and field visits to drug shops, from which field reports were derived. The analysis of data involved extraction and linking information on identified themes including “stakeholder mapping, practices, policy challenges, recommendations”, pre-defined key themes such as challenges faced by drug supply chain actors and practices in drug management. These reports also served as a basis to map the drug supply chain and document issues related to drug management.

#### Quantitative Analysis

The quantitative surveys consisted of the veterinary drug profiling with retailers and the survey on knowledge, practices, and awareness of veterinary practitioners about AMR and their related practices.

For the drug profiling, data obtained was recorded in MS excel to perform descriptive statistics. Generic drug names and active ingredients were used to classify the drugs into antibiotic, anthelminthic, arachnicide, vaccine, vitamin and iron supplement, antiprotozoal, disinfectant, anti-inflammatory and microbial supplement. Antibiotics were classified following OIE guidelines (11) into aminoglycosides, penicillins, quinolones, macrolides, polypeptides, sulphonamides, and tetracyclines.

For the knowledge, practice and awareness survey with veterinary practitioners, data was exported from the ODK server to STATA 15 (StataCorp) for further analysis. Descriptive statistics (frequencies and proportions) were carried out and whenever relevant, between districts comparisons were done using chi-square test. To analyse factors associated with appropriate knowledge of AMR by veterinary practitioners, a univariable analyse and a backwards stepwise selection Generalized Linear Models (GLM) was used. Each knowledge related question (Appendix 1) was independently analyzed by assigning a score to responses, either one (correct or appropriate response) or zero (incorrect or non-appropriate response). The scoring was done by the first author who is a veterinarian. To analyse how veterinary practitioners performed in the knowledge of AMR, the sum of each participant's answers was calculated. Those whose answered 70% or more correct were deemed to have good knowledge of antibiotics and AMR. Covariates with a *p* < 0.05 were included in the final multivariable analysis using GLM with a Poisson log linear link function, which was run in Stata package (StataCorp) version 14 to test the effect of different factors on the outcomes of interest (knowledge of AMR). A two-sided *p* < 0.05 was considered statistically significant.

## Results

### Qualitative Assessment and Description of the Veterinary Drug Supply Chain in Uganda

#### Map of the Veterinary Drug Supply Chain

**Figure 1:**
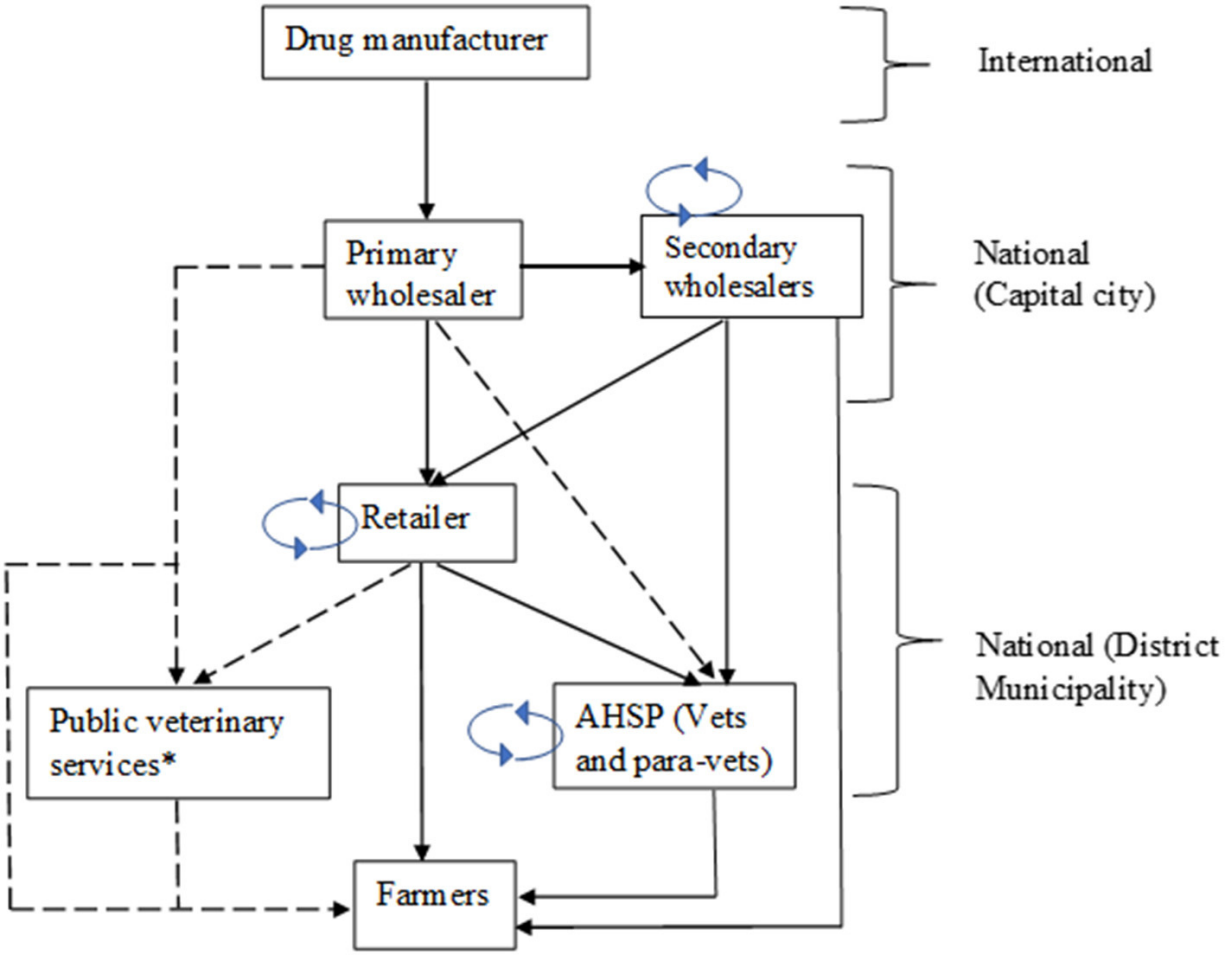
Veterinary drug and vaccine supply chains in Uganda. The flux distribution was determined based on perception of stakeholders (Full line = high flux distribution channel; Dotted line = low flux distribution channel); *Public veterinary services procure mainly vaccines for endemic diseases and acaricides; Continuous loop indicated the back-and-forth sales among same stakeholders. The determination of the flux was based on the knowledge and perception of stakeholders.

The supply of veterinary drugs in Uganda is made up of several actors who play distinct but complementary roles and who are mainly from the private sector. Drug manufacturers are the first level of actors (Figure 1). They are mostly based abroad and include international reputed pharmaceutical companies which have established a market in Africa. The primary wholesalers (or drug importers/distributors) are in the country and they hold large-scale business dealing directly with manufacturers. They supply drugs to secondary wholesalers and retailers, and to some extent to AHSPs and to the government. The secondary wholesalers hold medium scale businesses and mainly redistribute drugs to retailers (also called drug stockists) and to AHSPs such as veterinarians and para-veterinarians, who supply to farmers some of whom practice self-medication. Primary and secondary wholesalers have their main branches located in the capital city in Kampala. Drug retailers are located in the regions (or districts), and around municipalities from where they have access to infrastructures such as electricity. They mainly supply drugs to AHSPs and to livestock farmers. Drug retailers consider farmers as their first-choice customers because they pay higher prices as compared to AHSPs, who tend to bargain for lower prices as they have better market information of the products from wholesalers. Secondary wholesalers and retailers stock drugs based on demand forecasts, price, and profit margin. The transactions costs and their poor bargaining power may result in weak incentives for them to stock a wide variety of products or brands, leading to frequent shortage of products in the market. The government procures veterinary products for public use, especially livestock vaccines and acaricides, mainly from primary wholesalers.

The secondary wholesalers and retailers could buy drugs among themselves to accommodate market demand. This situation happens when a drug is scarce in some areas and is on excess in another area. The markets are constantly fluctuating, and products change hands many times. The determination of product prices at the supplier and the retailer nodes depends on the transaction costs incurred, mainly transport and storage. The distributors normally add a 15% charge for transport from the manufacturer to the primary wholesaler and a 30% mark-up (for distributor profit). The drug retailer stationed in the district adds a 30% for profit margin. At the farmer node, the cost of the drug is included in the service package of the AHSP.

The role of government in regard to regulation of veterinary drugs is divested to the NDA within the Ministry of Health. The NDA mandate includes supervision and control of importation, exportation of pharmaceuticals, promotion, and control of local drug production, as well as encouraging research and development of herbal medicines. Wholesalers and retailers hold a practice license provided by the NDA, which conducts routine monthly visits to drug dealers' premises to ensure compliance with regulations. The role of the Ministry of Agriculture, Animal Industry and Fisheries (MAAIF) is to provide extension and sensitization and capacity building of actors in the supply chain.

#### Challenges Reported by Drug Supply Chains Actors

The supply chain actors reported several constraints they face in the drug business. For example, repackaging of product in smaller units happens at different nodes of the supply chain. This practice can lead to product deterioration following exposure to abnormal temperature and sun light. There is weak enforcement of regulations such as quality control assurance which may accelerate proliferation of falsified or substandard products into the market. Actors reported that wholesalers do not have the same standard operating procedures for marketing drugs, as they all manage the product using own standards, which complicates quality control, thus absence of accountability if something goes wrong in the field including treatment failure or drug expiry. There is continuous questioning of farmers about the quality of drugs they receive since there are reported cases of poor effectiveness of some drugs after use. Regarding this, there is a blame game going on between retailers and farmers who accuse each other of being responsible for poor-quality drugs following poor storage and handling by retailers or inappropriate use by farmers.

Constraints reported by drug retailers include high transactions costs leading to high cost of the drugs, and consequently low profit margins particularly for small businesses that do not have sufficient financial capital. Uncontrolled transactions result in unethical competition, sidelining the small business owners, who then escape quality control.

Furthermore, farmers' fixed mindset does not enable smooth transition to accommodate new drugs in the market and is a barrier to drug uptake. For instance, farmers usually stick to known brands and are not flexible to change even though the active ingredient are the same. This situation prevents new drugs from penetrating the market, resulting in a limited range of options to farmers. Limited financial and human resources by authorities in charge of regulation of the drug supply chain were reported as a major challenge. According to stakeholders, inappropriate implementation of policies has led to lack of incentive of actors to comply to regulation. For example, lack of supportive measures such as compensation after drug confiscation by NDA makes some drug users not to report cases of drug expiry. Therefore, there has been an influx of substandard quality drugs in the market. The poor-quality drug or “fake drug” phenomenon was commonly pointed out by all actors as being a major issue. This is when a drug comes from a suspicious origin (e.g., unknown dealer to the community), or when farmers are deceived by health workers of treating non-curable diseases such as African swine fever. According to stakeholders, self-medication has gained ground, following poor access to quality drugs by farmers. It is therefore difficult to situate the responsibility of poor quality or “fake drug” when there is no traceability of products.

The actors recognize that there are gaps in the existing policy governing delivery of veterinary drugs, hence such a policy ought to be revised. A major gap identified is the poor collaboration between government bodies who play important roles in the delivery of drugs, and the laborious protocols and procedures for registering new drugs, leading to stakeholders taking “shortcuts” to release the product in the market. The actors also stressed the need to set-up regulatory authorities/bodies to track unqualified personnel. Major challenges reported by regulators include lack of personnel at district level; and lack of laboratory capacity for quality control especially for identifying fake drugs. They also mentioned that products licensed by NDA are of good quality and are efficient however drug stockists reduce the efficiency of the drugs through their practice. Poor practices of drug stockists include reconstitution of vaccines for retail sale or dividing the vaccine capsules; sale of expired drugs to farmers by changing labels to extend the shelf life of drugs or altering the expiry dates and also repackaging drugs. Therefore, drug shops are limited on the amount of stock at a given time as well as bulk packs. They further stated that the problem was not the drugs but the government policy of liberalization where everyone can run a drug shop for the money and not as a form of service.

### Veterinary Drug Profiling and Drug Sale Practices

#### Sociodemographic Characteristics of Drug Retailers

Most drug retailers interviewed in Mukono and Lira were male, holding either a diploma or a lower academic qualification (Table 2).

**Table 2:** Demographic characteristics study participants (drug retailers) by district.

**Variable**	**Category**	**Lira (*n =* 10)**	**Mukono (*n =* 13)**
Gender of respondent	Male	8 (80%)	7 (54%)
	Female	2 (20%)	6 (46%)
Academic qualification	Bachelor of Science veterinary medicine	1 (10%)	1 (8%)
	Diploma in animal production^*^	4 (40%)	9 (69%)
	Certificate in animal production^*^	3 (30%)	2 (15%)
	Primary education	1 (10%)	0 (0%)
	Unknown	1 (10%)	1 (8%)

* *Diploma and certificate programs help prepare students for higher education and advance their careers. They are usually short in length since they are intended to cover specific areas*.

#### Category of Antibiotics Stocked by Retailers

**Figure 2:**
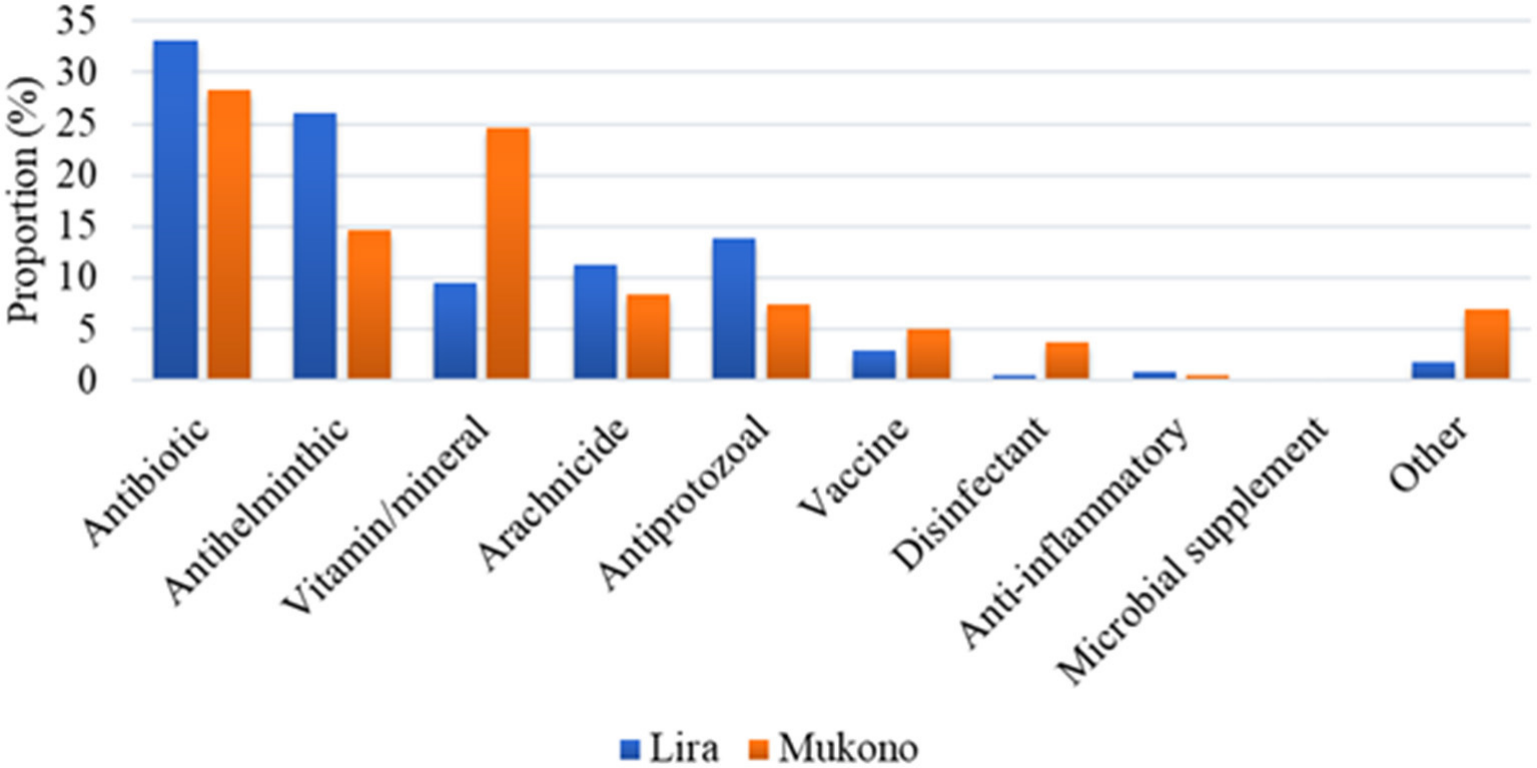
Drugs and supplies/biologicals stocked in shops in Lira and Mukono districts. Other Category Includes Growth Promoters, Feed Additives, Detergents, Sedatives.

In total 1,172 drug brand names were recorded from the 23 drugs retail shops in districts of Lira, (29%) and Mukono (71%). Antibiotics (33%) and anthelminthics (26%) were the most stocked drugs by retailers in Lira district, while antibiotics (28%) and vitamins/minerals (25%) represented the most stocked drugs by retailers in Mukono district (Figure 2).

The most common classes of antibiotics recorded in both districts were tetracyclines (53%) and sulphonamides (18%), followed by macrolides (9%) and quinolones (7%). Polypeptides and aminoglycosides were the least frequently recorded group of antibiotics and were only found in Mukono. Within the class of tetracyclines, oxytetracycline was the most frequently encountered antibiotic compound; while sulfadimidine was the most frequently encountered antibiotic compound within the class of sulphonamides. All antibiotic compounds, except tylosin, were reported to have been in the market for at least 15 years, with oxytetracycline and procaine penicillin being the oldest with 30 years. Tylosin was reported to be the newest antibiotic substance on the market, available since about 8 years (Table 3).

**Table 3:** Classes of antibiotics stocked in retail shops in Lira and Mukono districts.

**Antibiotic class**	**Lira, *n =* 114**	**Mukono, *n =* 234**	**Total, *n =* 348**	**Antibiotic compound per antibiotic class**
Tetracycline	67 (59%)	118 (50%)	185 (53%)	Oxytetracycline, 93% Oxytetracycline cocktail^**^, 6% Doxycycline, 1%
Sulphonamide	18 (16%)	44 (19%)	62 (18%)	Sulfadimidine, 32% Trimethoprim + Sulfamethazine, 29% Trimethoprim + Sulfadiazine, 27% Sulfamonomethoxine, 7% Sulphadimidine cocktail^*^, 5%
Macrolide	7 (6%)	23 (10%)	30 (9%)	Tylosin, 80% Erythromycin 20%
Quinolone	3 (3%)	20 (9%)	23 (7%)	Enrofloxacin, 87% Flumequin, 13%
Penicillin + Aminoglycoside	10 (9%)	9 (4%)	19 (5%)	Procaine penicilin + Dihydrostreptomycin, 83% Procaine penicilin + Neomycin + Streptomycin, 17%
Penicillin	6 (5%)	9 (4%)	15 (4%)	Cloxacillin benzathine, 92% Amoxicillin, 8%
Aminoglycoside	3 (3%)	9 (4%)	12 (3%)	Gentamicin, 75% Neomycin, 25%
Polypeptide	0 (0%)	2 (1%)	2 (1%)	Colistin, 100%

* *Sulphadimidine + diaveridine, excipient, vit K*.

** *Oxytetracyclin + hydrochloride, neomycin sulfate, Vits A, D3, E B1,2, 6,12 K3, C, and Oxytetracyclin + nicotinamide, folic acid, methionine, lysine or Vits A, D3, E, K3, B2, B 12*.

According to the drug producer recommendations, the main routes of administration of antibiotics to livestock as recorded from the package were oral (51%), followed by intramuscular injection (15%) and topical application (13%). Other administration routes include sub-cutaneous, intra-uterine, intramammary, eye drop and intravenous.

#### Practices of Drug Retailers and Factors Influencing Drug Choice by Buyers

From the retailer's point of view, there is no restriction to whom they sell drugs to, and most of the time, buyers (mostly farmers and veterinary practitioners) ask for a drug they want by its trade name. This is influenced by their experience with these drugs or the duration of use of the drugs (usually those that have been on the market for long). Farmers mostly stick to the drugs they know and have been using in the past; they are reluctant to change to drugs that are new in the market. The cost of the drug was also mentioned as a factor that influences choice of the buyer, as some buyers weigh prices of different drugs and opt for the cheapest option. Location (drug shop close to client) was an important factor, and we observed that the urban shops received the most clients. Perceived effectiveness of the drug experienced by the buyer was another factor affecting drug purchase. It was reported that farmers communicate drug success or failure and recommend drugs that work to each other. When a buyer comes without prior knowledge, recommendations are made by the retailer based on the buyer's description of the clinical signs of the disease. From a AHSP perspective, the choice of a drug takes into account the administration mode of the drug (easier to administer, not requiring lot of restraining as in the case of injectables), the preference of the farmer (single dose not requiring follow-up), the cost of the drug (affordable for the farmer). Given that most of the times there is no laboratory diagnostics carried out prior to treatment, ASHPs prefer broad spectrum anthelminthics such as ivermectin (targets internal and external parasites), or antibiotics such as oxytetracycline. Aliquoting drugs into small doses is common for buyers who cannot afford to buy the whole pack. This is carried out using syringes, empty drug containers, including empty vials dedicated for human drugs which are used to aliquot mostly injectable drugs.

Drug retailers noted that there is continuous advice to buyers about usage of drugs (dosage or usage frequency and duration of usage), especially for arachnicides and poultry drugs that require mixing or dilution before use. Most drug shops had a certificate of operation on display with the respective qualifications mentioned. However, the person whose qualification is displayed was most of the times not the person interviewed, nor the one doing the sales. We also noticed that drug shops are sometimes run as family business and a family member with no qualifications in animal health or animal production is involved in the sale of drugs. This helps cut expenses and probably tackles unemployment given the high unemployment rates in the area. Another scenario is that a person without specific veterinary sciences training is sometimes hired. It is also common for agricultural shops to get involved in the sale of veterinary drugs, although they are not licensed by the NDA. They sell “fast moving” drugs like oxytetracyclines, acaricides and wound sprays. It is however unlikely that someone would see these drugs as they are kept away from display and only brought out when a trustworthy client asks for them.

### Survey With Veterinary Practitioners on Knowledge, Practices, and Awareness About AMR and Perception of Related Policies

#### Demographics and Services Delivered by Veterinary Practitioners Use

Majority of veterinary practitioners who took part in the survey were male mainly offering disease treatment services only. Most of them hold a diploma and have been practicing for at least 5 years (Table 4).

**Table 4:** Demographic characteristics of veterinary practitioners.

**Variable**	**Category**	**Lira (*n =* 32)**	**Mukono (*n =* 68)**
q8-Gender of respondent	Male	29 (91%)	59 (87%)
	Female	3 (9%)	9 (13%)
q10-Nature of the business	Practicing treatment only	29 (91%)	49 (72%)
	Practicing treatment and drug retail shop	3 (10%)	19 (28%)
q11-Years in business	0–1 year	1 (3%)	5 (7%)
	2–4 years	9 (28%)	12 (18)
	5–10 years	14 (44%)	24 (35%)
	More than 10 years	8 (25%)	27 (40%)
q12-Academic qualification	Bachelor of Veterinary Medicine	1 (3%)	4 (8%)
	Bachelor of Science	0 (0%)	6 (6%)
	Diploma	21 (66%)	35 (51%)
	Certificate	5 (16%)	20 (29%)
	High school	1 (3%)	2 (3%)
	Primary school	4 (12%)	1 (2%)

Antibiotics were mentioned to be the most profitable drug category for veterinary practitioners (Lira, 81% and Mukono, 59%). In both districts, practitioners dealt mostly with cattle and pigs. Practitioners who dealt with poultry were only registered in Mukono district. Most veterinary practitioners provided drugs to small scale farmers (Lira, 84%; Mukono, 73%) (Table 5).

**Table 5:** Characteristics of business delivered to farmers by veterinary practitioners.

**Variable**	**Category**	**Lira (*n =* 32)**	**Mukono (*n =* 68)**
q17-Drug category important for the business	Antibiotics	26 (81%)	40 (59%)
	Antihelmintics	5 (16%)	18 (26%)
	Arachnicides	0 (0%)	3 (4%)
	Vaccines	1 (3%)	7 (10%)
	Vitamins	0 (0%)	0 (0%)
q13-Livestock dealt with most	Cattle	27 (84%)	34 (50%)
	Pigs	5 (16%)	20 (29%)
	Poultry	0 (0%)	13 (19%)
	Sheep/goats	0 (0%)	1 (1%)
q16-Customer to veterinary practitioners	Small scale farmer	26 (84%)	49 (73%)
	Large scale (commercial) farmers	5 (16%)	18 (27%)

Most veterinary practitioners reported administering drugs to animals or selling drugs to farmers without prescription (Lira, 37%; Mukono, 40%). They mostly decided on drugs based on symptoms as described by the farmer and verified by themselves (Lira, 75%; Mukono, 78%), or based on their own judgement following a farmer's explanation without seeing the animal (Lira, 25%; Mukono, 21%). Dosage was generally determined following instructions on the drug packaging, while weight of the animal was estimated by the farmer or the practitioner. According to practitioners, most farmers in Lira (87%) administered the drugs to animals by themselves. Drugs were mostly sold as single tablets by 25% of practitioners in Mukono, while 65% of practitioners said they provide enough drugs for the whole course of treatment with follow-up visits. There were complaints from several clients (farmers) about treatment failure (Lira, 44%; Mukono, 74%). Arachnicides/acaricides were reported to be the drug that failed most, followed by antibiotics and antihelminthics (Table 6).

**Table 6:** Practices of veterinary practitioners in drug use.

**Variable**	**Category**	**Lira (*n =* 32)**	**Mukono (*n =* 68)**
q21-Sale of drugs to farmers	On prescription only (from another veterinary practitioner)	20 (63%)	41 (60%)
	Both with and without prescription	12 (37%)	27 (40%)
	Without prescription	0 (0)	0 (0%)
q24-Basis for deciding to administer or sell antibiotic to farmers	Symptoms as explained by the farmer and verified by veterinary practitioner	24 (75%)	53 (78)
	Laboratory test results provided by farmer or done by veterinary practitioner	0 (0%)	1 (1%)
	Own judgement following farmers' explanation (without seeing the animal)	8 (25%)	14 (21%)
q25-Estimation of dosage	As indicated on the drug packaging	13 (41%)	21 (31%)
	Own judgement based on experience of success	4 (12%)	26 (38%)
	Estimated weight of the animal by farmer or practitioner	15 (47%)	20 (29%)
	Other	0 (0%)	1 (1%)
q27-Way of administering the drug to animals	Single dose/one-time measurement depending on the farmers' capacity	2 (6%)	17 (25%)
	Whole package for whole course of treatment	2 (6%)	44 (65%)
	As indicated on drug packaging	0 (0%)	1 (1%)
	I don't administer^*^	28 (87%)	6 (9%)
q28-Drug failure reported by customers (farmers)	Yes	14 (44%)	50 (74%)
	No	18 (56%)	18 (26%)
q30-mnagement of expired drugs	Discard	29 (91%)	56 (84%)
	Never experienced	2 (6%)	2 (4%)
	Return to National Dru Authority	0 (0%)	1 (1%)
	Return to the wholesaler	1 (3%)	7 (10%)
	Sell to clients at cheaper price	0 (90%)	1 (1%)

* *These categories of animal health practitioners sell the drug to farmers who administer by themselves*.

#### Awareness and Knowledge of Veterinary Practitioners About AMR

Most veterinary practitioners had heard about AMR (Lira, 75%; Mukono, 79%) mainly through one-to-one communication among practitioners (50% and 35%, respectively, in Lira and Mukono). Other important awareness channels include during training sessions organized by government, by development organizations, broadcasting (radio and television), newspapers, and internet. The role of antibiotics was moderately understood, with majority of practitioners correctly indicating that antibiotics are effective in managing bacterial infections (Lira, 72%; Mukono, 75%). Knowledge about antibiotic residues in animal source foods such as meat and eggs and knowledge about withdrawal periods and processes of acquiring antibiotics residues through food products was very high. However, the ways humans can acquire resistance to antibiotics was not well-understood (Table 7).

**Table 7:** Knowledge of veterinary practitioners about roles of antibiotics and antibiotic resistance.

**Variable**	**Category**	**Lira, *n =* 32**	**Mukono, *n =* 68**	***P*-value**
**Awareness about antibiotic resistance**				
q31-Heard about antibiotic resistance phenomenon	Yes	24 (75%)	54 (79%)	0.619
	No	8 (25%)	14 (21%)	
q32-antibiotic resistance awareness channel	Learned about AMR from my background training	8 (34%)	22 (41%)	0.615
	Heard from radio	2 (8%)	2 (4%)	
	Learnt from a colleague	12 (50%)	19 (35%)	
	Learnt from a short training/workshop	2 (8%)	9 (16%)	
	Red from newspaper	0 (0%)	1 (2%)	
	Other	0 (0%)	1 (2%)	
**Drivers of antibiotic resistance**				
q37-Antibiotic resistance is caused by using antibiotics when not indicated	Agree	19 (59%)	49 (72%)	0.205
	Disagree	13 (41%)	19 (28%)	
**Roles of antibiotics**				
q38-Antibiotics are effective in managing bacterial infections	Agree	23 (72%)	51 (75%)	0.740
	Disagree	9 (28%)	17 (25%)	
q39-Antibiotics are effective in managing viral infections	Agree	4 (12%)	30 (44%)	0.002^**^
	Disagree	28 (88%)	38 (56%)	
q40-Antibiotics are effective in managing protozoal infections	Agree	27 (84%)	49 (72%)	0.179
	Disagree	5 (16%)	19 (28%)	
q41-Antibiotics are effective in managing parasitic infections	Agree	6 (19%)	5 (7%)	0.089
	Disagree	26 (81%)	63 (93%)	
q45-Antibiotics are effective in managing pain and inflammation	Agree	27 (84%)	53 (78%)	0.453
	Disagree	5 (16%)	15 (22%)	
q43-Antibiotics are effective in boosting animal growth	Agree	25 (78%)	19 (28%)	0.000^**^
	Disagree	7 (22%)	49 (72%)	
**Antibiotic residues**				
q44-Residues of antibiotics can be found in meat	Agree	31 (97%)	61 (88%)	0.218
	Disagree	1 (3%)	7 (10%)	
q45-Residues of antibiotics can be found in milk	Agree	31 (97%)	59 (87%)	0.116
	Disagree	1 (3%)	9 (13%)	
q46-Residues of antibiotics can be found in eggs	Agree	26 (81%)	51 (75%)	0.488
	Disagree	6 (19%)	17 (25%)	
**How is antibiotic resistance acquired**				
q47-People can acquire resistance through consuming animal products that contain residues of antibiotics	True	31 (97%)	59 (87%)	0.231
	False	1 (3%)	4 (13%)	
q48-People can acquire resistance through direct bodily contact with sick animals	True	12 (37%)	32 (47%)	0.279
	False	20 (63%)	36 (53%)	
q49-People can acquire resistance through contact with animal feces	True	20 (62%)	43 (63%)	0.472
	False	12 (36%)	25 (34%)	
q50-People can acquire resistance through body fluids of sick animals	True	29 (91%)	57 (84%)	0.585
	False	3 (9%)	11 (16%)	
**Withdrawal periods**				
q53-Observance of withdrawal period makes the animal product safer for human consumption	Agree	31 (97%)	63 (94%)	0.086
	Disagree	1 (3%)	4 (6%)	

** *significant at P < 0.05*.

#### Factors That Influence Knowledge of Veterinary Practitioners About Antibiotics and AMR

Awareness of veterinary practitioners about AMR, district of origin (Lira) and category of clients (small scale farms) had a positive effect on knowledge of veterinary practitioner about AMR (Table 8).

**Table 8:** Factors associated with knowledge of veterinary practitioner about antibiotics and AMR.

Poisson regression		Number of obs. = 65				
Wald chi2(9) = 48.58		Prob > chi2 = 0.0000				
Pseudo R2 = 0.0374		Log pseudolikelihood = −122.00191				
**Variable**	**Coef**.	**Robust Std. Err**.	**z**	**P>z**	**[95% confidence interval]**	
**Determination of dosage by farmer**						
Own judgment						
As indicated on the drug pack	0.092218	0.0528942	1.740	0.081	−0.01145	0.195889
**Heard about AMR**						
No^*^						
Yes	0.2543311	0.0922078	2.760	0.006^**^	0.073607	0.435055
**Category of clients**						
Small scale farms^*^						
Large scale farms	−0.1404909	0.0595735	−2.360	0.018^**^	−0.25725	−0.02373
**Level of education of veterinary practitioners**						
Primary^*^						
Diploma	0.0640564	0.06002	1.070	0.286	−0.05358	0.181693
BVM	0.119419	0.0726594	1.640	0.100	−0.02299	0.261829
**Most important drugs in the business**						
Antihelminthics^*^						
Antibiotics	−0.0215451	0.0625481	−0.340	0.731	−0.14414	0.101047
Arachnicides/vaccines	0.1090097	0.0792294	1.380	0.169	−0.04628	0.264297
**District of operation**						
Mukono^*^						
Lira	0.2139437	0.0693367	3.090	0.002^**^	0.078046	0.349841
**Most frequent way of administering the drug to animals**						
Whole course of treatment as recommended^*^						
Single dose/one-time application	−0.0347081	0.0525027	−0.660	0.509	−0.13761	0.068195
_cons	1.441464	0.0934914	15.420	0.000	1.258224	1.624704

* *reference*;

** *significant at P < 0.05*.

#### Perception of Veterinary Practitioners About Critical Actions to Mitigate AMR

Many veterinary practitioners were not conversant about veterinary drug policies of the country, with Mukono having a higher number (72%) compared to Lira (37%). However, the most urgent capacity needs according to practitioners in Lira were a better understanding of the policies about the use of veterinary drugs in the country and a better knowledge on how to use AMs in livestock. While in Mukono, the most urgent capacity needs were the understanding of the mechanism of AMR and a better knowledge on how to use AMs in livestock. Perception of veterinary practitioners about critical actions for the sustainable control of AMR include raising awareness of farmers about the impact of misuse of antibiotics; re-enforcement of disease control measures in livestock and stronger and directed policies on AM use (Table 9).

**Table 9:** Actions needed to control AMR according to veterinary practitioners.

**Variable**	**Category**	**Lira**	**Mukono**
q55-I am conversant about the veterinary drug policy of Uganda	Agree	20 (63%)	19 (28%)
	Disagree	12 (37%)	49 (72%)
	Total	32 (100%)	68 (100%)
q59-Urgently needed to mitigate AMR	Knowledge on how to use AMs	8 (25%)	15 (22%)
	Understand mechanisms of AMR	6 (19%)	29 (43%)
	Knowledge on when to prescribe AMs	3 (9%)	5 (7%)
	Understand links between the health of humans, animals and the environment	5 (16%)	7 (10%)
	Understand the policies about the use of veterinary drugs in the country	10 (31%)	12 (18%)
	Total	32 (100%)	68 (100%)
q60-Critical actions for the sustainable control of AMR	Stronger and directed policies on AM use	10 (31%)	35 (52%)
	Raise awareness of farmers about the impact of AM misuse	13 (41%)	24 (35%)
	Strict monitoring of drug import into the country	2 (6%)	4 (6%)
	Re-enforce disease control in livestock	5 (16%)	1 (2%)
	Enhance disease diagnostic in livestock	0 (0%)	1 (2%)
	Strengthen quality control of drugs stocked in the country	2 (6%)	3 (4%)
	Total	36 (100%)	78 (100%)

## Discussion

The veterinary drug business in Uganda is largely driven by private sector and is characterized by a diversity of actors who play distinct but complementary roles. The drug business is operated by personnel with sometimes limited training in drug management, which is in line with findings of Ilukor et al. (3) and Byarugaba (4) who reported that the animal health sector in Uganda has a high percentage of non-trained service providers. In most veterinary drug sale points, drugs can be accessed by anyone (veterinary practitioners or not), regardless of what drug is needed. This situation reflects the poor regulation of the veterinary drug market. Lack of traceability of drugs was widely pointed out by stakeholders as a major challenge leading to proliferation of “fake drugs” in the market. According to Granados-Chinchilla and Rodríguez (12), the Sub-Saharan African market is highly affected by counterfeit veterinary drugs. Though these counterfeit and non-compliance of drugs can induce adverse effects during their utilization, there is no monitoring system of veterinary medicines (13).

We found that all antibiotics (except tylosin) stored in drug shops have been on the market for at least 15 years, suggesting constant and wide use of these drugs in livestock. This situation also points to the limited diversity in the antibiotic classes marketed, with oxytetracycline and sulfadimidine being the common antibiotics accessed by farmers for many years. A study in Ghana, reported the same antibiotics to be commonly used in livestock in many smallholder livestock (14). Tetracyclines are a family of compounds frequently employed due to their broad spectrum of activity as well as their low cost, compared with other antibiotics. Currently, there are over 20 tetracyclines available; however, tetracycline, chlortetracycline, oxytetracycline, and doxycycline are the most common ones in veterinary medicine (15). In addition to therapeutic purposes, in many other countries, tetracyclines are often incorporated into livestock feed at subtherapeutic doses as growth promoters for swine and poultry and in aquaculture (12).

The lack of diversity in the use of drugs reflects the low level of sophistication of the drug market in Uganda, which is likely linked to the limited disease diagnostic capacities that prevail, hence restricting the choice of drugs, therefore leading to the constant use of broad-spectrum antibiotics more frequently. However, this lack of diversity in the use of drugs could also be a good thing as it reduces the risk of multidrug resistance to several classes of antimicrobials; considering the fact that that excessive use of antibiotics in humans leads to emergence of resistant organisms (16). The criteria for choosing a drug by veterinary practitioners which were mainly based on their experience with the drug was rather subjective; hence the change of giving a wrong treatment to animal is high. This is exacerbated by the fact that in most African countries such as Uganda, there inappropriate limited diagnostic facilities including antimicrobial sensitivity testing (10). Previous studies in Uganda by Dione et al. (17) and Ilukor and Birner (18) reported that incorrect diagnosis, under-dosing and overdosing and wrong drug administration routes, poor handling and storage of drugs were common practice among farm households and service providers in pig and cattle production systems, respectively. In fact, service providers were found not to be able to prescribe correct drugs for treatment of specific cattle diseases (18). Although the link between stakeholder practices and AMR is still weak, according to Ayukekbong et al. (10), the lack of appropriate quality control regulations as reported in the distribution of veterinary of drugs including antimicrobials could be a contributing factor to the misuse of antimicrobials; consequently, any imprudent practice along drug supply chain may fuel the emergence of resistance.

Pig/poultry and cattle farmers were the main customers of the drug, in Mukono and Lira districts, respectively. This can be explained by the difference in livestock production systems between the two districts: Lira district has a more rural production system with more cattle-farming, compared to Mukono which is characteristic of a peri-urban farming with increasing poultry and pig production. There was no market for antibiotics in small ruminant production in the studied districts. This could be explained by the fact that small ruminants are less market oriented; hence they are kept in low input systems with low investment of farmers on drugs and other inputs such as feed.

High awareness of veterinary practitioners about antibiotic residues in animal products and the importance of drug withdrawal time was reported in the study. However, it was not clear if practitioners advised farmers accordingly. The fact that there is lack of effective monitoring system for drug residues along the food supply chain in Uganda, makes it difficult to assess eventual risks to consumers. However, it is important to note that the profile of antibiotics reported in our study such as tetracyclines, sulfamidines and sulfamethoxazole-trimethoprim matches those of AMR reported in several studies in Uganda (19–21). Low resistance was reported in Uganda for the less commonly accessible antibiotics reported in our study such as ciprofloxacin and gentamicin (19, 21). Furthermore, another study (19) showed that AMR correlated negatively with the local price of the antibiotic, with the most expensive antibiotics (nalidixic acid and ciprofloxacin) showing near-zero resistance. These findings are in line with ours where accessible antibiotics are those that are said to be more affordable.

Awareness about AMR is an important factor identified for understanding the roles of antibiotics among veterinary practitioners, as is education. Both drug retailers and veterinary practitioners operate in an environment, which seems to be driven by financial profit, rather than quality of service to the end-users. This is aggravated by the low purchase capacity of farmers who aim to optimize investment for financial return from the farm. Therefore, quality of products seems not to be a focus, especially when regulators do not have a full hand on this. According to Byarugaba (4), the weaknesses in the implementation of policies are a challenge to sustainable drug resistance control and prevention of AMR as these laws exist only on paper or are poorly communicated to the stakeholders and also their implementation may be difficult due to poor funding. A possible underlying cause of this could be the lack or inadequate consultation when developing the policies. While the need for an improved policy environment of veterinary drug management is urgent, resulting policies and regulations should not undermine the business capacities of input suppliers, but the focus should be on increasing their knowledge on AMR and clearly defining their roles in supporting prevention of AMR.

## Conclusion

Our study investigated the veterinary drug supply chain, the knowledge, practices, awareness and practices of veterinary practitioners on antimicrobial usage, and the related policy landscape. The common classes of antibiotics recorded in both districts were tetracyclines. Stakeholders of the drug supply chain pointed out lack of traceability of products as a major contributor to poor quality of drugs found in the market. Potential drivers of misuse of antibiotics, include low level of education of actors such as drug retailers, veterinary practitioners, poor handling of drugs at purchase and administration practices, low enforcement of policy and regulations and lack of awareness of stakeholders about policies that regulate use of drugs. Thus, future interventions to reduce misuse of drugs in small-scale livestock production systems should target improvement of the business of veterinary drug input suppliers, and deliberately involve a strong policy advocacy component.

## Data Availability Statement

The raw data supporting the conclusions of this article will be made available by the authors, without undue reservation.

## Ethics Statement

The studies involving human participants were reviewed and approved by Uganda National Committee for Scientific Technology with approval reference number A583. The patients/participants provided either their oral (informal interviews) or written informed consent to participate in this study.

## Author Contributions

MD and BW conceived the study. MD, WCA, and BW developed the data collection tools. MD and WCA collected the data. MD analyzed the data and wrote the first draft of the manuscript. BW, EO, and FE made critical review and edits to the final draft. All authors contributed to the article and approved the submitted version.

## Funding

We also acknowledge funding of the CGIAR Research Program (CRP) on Livestock and thank donors and organizations which globally support its work through their contributions to the CGIAR Trust Fund.

## Conflict of Interest

The authors declare that the research was conducted in the absence of any commercial or financial relationships that could be construed as a potential conflict of interest.

## Publisher's Note

All claims expressed in this article are solely those of the authors and do not necessarily represent those of their affiliated organizations, or those of the publisher, the editors and the reviewers. Any product that may be evaluated in this article, or claim that may be made by its manufacturer, is not guaranteed or endorsed by the publisher.

## References

[1] Beyene T. Veterinary drug residues in food-animal products: its risk factors and potential effects on public health. J Veterinar Sci Technol. (2016) 7:1. 10.4172/2157-7579.1000285

[2] Dar OA Hasan R Schlundt J Harbarth S Caleo G Dar FK. Exploring the evidence base for national and regional policy interventions to combat resistance. Lancet. (2016) 387:285–95. 10.1016/S0140-6736(15)00520-6 26603921

[3] Ilukor J Birner R Rwamigisa PB Nantima N. The provision of veterinary services: who are the influential actors and what are the governance challenges? A case study of Uganda. Exp Agric. (2015) 51:408–34. 10.1017/S0014479714000398

[4] Byarugaba DK. A view on antimicrobial resistance in developing countries and responsible risk factors. Int J Antimicrob Agents. (2004) 24:105–10. 10.1016/j.ijantimicag.2004.02.015 15288307

[5] Sakeena MHF Bennett AA McLachlan AJ. Enhancing pharmacists' role in developing countries to overcome the challenge of antimicrobial resistance: a narrative review. Antimicrob Resist Infect Control. (2018) 7:63–63. 10.1186/s13756-018-0351-z 29744044 PMC5930749

[6] Muloi D Fèvre EM Bettridge J Rono R Ong'are D Hassell JM. A cross-sectional survey of practices and knowledge among antibiotic retailers in Nairobi, Kenya. J Glob Health. (2019) 9:010412. 10.7189/jogh.09.020412 31489183 PMC6708591

[7] UBOS. National Population and Housing Census (2014). Provisional results. November (2014). Revised edition, Uganda Bureau of Statistics, Kampala, Uganda (2014).

[8] UBOS MAAIF. The National Livestock Census Report by the Ministry Of Agriculture, Animal Industry And Fisheries, Entebbe, Uganda and the Uganda Bureau of Statistics. Kampala (2009).

[9] Ouma EA Dione M. Uganda Smallholder pig Value Chain Site Scoping Report: Lira, Kibaale and Hoima districts. Nairobi: ILRI (2014).

[10] Ayukekbong JA Ntemgwa M Atabe AN. The threat of antimicrobial resistance in developing countries: causes and control strategies. Antimicrob Resist Infect Control. (2017) 6:47. 10.1186/s13756-017-0208-x 28515903 PMC5433038

[11] OIE. OIE List of Antimicrobial Agents of Veterinary Importance. (2015). Available online at: http://www.oie.int/fileadmin/Home/eng/Our_scientific_expertise/docs/pdf/Eng_OIE_List_antimicrobials_May2015.pdf (accessed June 20, 2021).

[12] Granados-Chinchilla F Rodríguez C. Tetracyclines in food and feedingstuffs: from regulation to analytical methods, bacterial resistance, and environmental and health implications. J Anal Methods Chem. (2017) 2017:1315497. 10.1155/2017/1315497 28168081 PMC5266830

[13] Mouiche MMM Njingou BZN Moffo F Mpouam SE Feussom JMK Awah-Ndukum J. Veterinary pharmacovigilance in sub-Sahara Africa context: a pilot study of adverse reactions to veterinary medicine in Cameroon. BMC Vet Res. (2019) 15:301. 10.1186/s12917-019-2043-1 31426790 PMC6701137

[14] Sekyere OJ. Types and selling practices of antibiotics in veterinary shops in Ashanti Region, Ghana. Int J Food Agric Veterinary Sci. (2014) 4:87–96. Available online at: http://cibtech.org/J-FOOD-AGRI-VETERINARY-SCIENCES/PUBLICATIONS/2014/Vol_4_No_2/JFAV-017-022-JOHN-TYPES-GHANA.pdf

[15] Fritz JW Zuo Y. Simultaneous determination of tetracycline, oxytetracycline, and 4-epitetracycline in milk by high-performance liquid chromatography. Food Chem. (2007) 105:1297–301. 10.1016/j.foodchem.2007.03.04731637854

[16] Goossens H. Antibiotic consumption and link to resistance. Clin Microbiol Infect Suppl. (2009) 3:12–15. 10.1111/j.1469-0691.2009.02725.x 19366364

[17] Dione M Ouma EA Lule P Pezo D. Animal health services delivery systems and disease surveillance in the smallholder pig value chain in Uganda. In: 2nd Animal International Conference on Animal Health Surveillance. La Havana (2014).

[18] Ilukor J Birner R. Measuring the quality of clinical veterinary services for Cattle: an application of a role play experiment in rural Uganda. BMC Res Notes. (2014) 7:894. 10.1186/1756-0500-7-894 25491745 PMC4295323

[19] Weiss DW Wallace RM Innocent B Rwego IB Gillespie TR Chapman CA. Antibiotic-resistant escherichia coli and class 1 integrons in humans, domestic animals, and wild primates in rural Uganda. Appl Environ Microbiol. (2018) 17:21. 10.1128/AEM.01632-18 30171005 PMC6193383

[20] Okubo T Yossapol M Maruyama F Wampande EM Kakooza S Ohya K. Phenotypic and genotypic analyses of antimicrobial resistant bacteria in livestock in Uganda. Transbound Emerg Dis. (2019) 66:317–26. 10.1111/tbed.13024 30260584

[21] Byarugaba DK Kisame R Olet S. Multi-drug resistance in commensal bacteria of food of animal origin in Uganda. African J Microbiol Res. (2011) 5:1539–48. 10.5897/AJMR11.202

