# Supplementary material for: Supply Chain and Delivery of Antimicrobial Drugs in Smallholder Livestock Production Systems in Uganda
Source: Front Vet Sci. 2021 Sep 8;8:611076. doi: 10.3389/fvets.2021.611076 (PMC8455991; doi:10.3389/fvets.2021.611076)
Supplement: Supplementary file 1 [file Table_1.DOCX]

**Tool for Assessing the knowledge, attitudes, practices of veterinary practitioners and drug prescribers on use of antimicrobials in livestock production systems**

Michel Dione, Christine Amia Winfred and Barbara Wieland

**Background**: Antimicrobial Resistance (AMR) has become a major public health challenge in most parts of the world and is a major threat to gain in bacterial disease control in both animals and humans. The role of prescribers in the control of antibiotics is identified as crucial in developing interventions to control AMR. To guide policy recommendations on control of AMR a study is planned among veterinary drug prescribers including private vets, para-vets and drug stockists to identify gaps in their knowledge attitude and practices of AMR and to document their prescription practices.

**Section A:** **Background information**

| 1. Questionnaire ID | |  | | |
| --- | --- | --- | --- | --- |
| 1. District | |  | | |
| 1. Sub-county | |  | | |
| 1. Village | |  | | |
| 1. Date of Survey | | DD/MM/YYYY | | |
| 1. Enumerator’s name | |  | | |
| 1. Name of the respondent | |  | | |
| 1. Sex of the respondent | | 🞏1 Male, 🞏2 Female | | |
| 1. Age of respondent (years) | |  | | |
| 1. Nature of work | | 🞏1 Practicing veterinary drug retail only  🞏2 Practicing treatment only  🞏3 Practicing both | | |
| 1. Number of year working in the business / in practice | | 1🞏- 0-1 2🞏- 2-4  3🞏- 5-10 4🞏- More than 10 | | |
| 1. Qualification of the respondent | | 🞏1 Bachelor of Veterinary Medicine (BVM)  🞏2 Bachelor of Science (BSc)  🞏3 Diploma  🞏4 Certificate  🞏5 High School  🞏6 Primary school | | |
| 1. Which livestock do you deal with most (single choice) | | 🞏1 Pigs  🞏2 Cattle  🞏3 Sheep/goats  🞏4 Poultry  🞏5 Camels | | |
| 1. What are the main (5) animal disease problems that you have treated / provided drugs for during the last 6 months (for each species) | | | | |
| 1. Pigs | 1. Cattle | 1. Sheep / goats | 1. Poultry | 1. Camels |
|  |  |  |  |  |
|  |  |  |  |  |
|  |  |  |  |  |
|  |  |  |  |  |
|  |  |  |  |  |

**Section B: Drug management practices and awareness about AMR**

| 1. To whom do you provide drugs to mostly?   **(drug stockists)** | 🞏1 Farmers  🞏2 Para-veterinarians  🞏3 Veterinarians  🞏4 Companies/ Organisations (Name)………………………………….  🞏5 Other (specify) | | | | | | | | | | | |
| --- | --- | --- | --- | --- | --- | --- | --- | --- | --- | --- | --- | --- |
| 1. To whom do you provide your services mostly?   **(practitioners)** | 🞏1 Small scale Farmers  🞏2 Commercial / Large scale farmers  🞏3 Other (specify) | | | | | | | | | | | |
| 1. Which drug category is most important for business / Practice? | 🞏1 Vaccines  🞏2 Anthelmintic (Dewormers)  🞏3 Arachnidicides (Ectoparasites)  🞏4 Antibiotics  🞏5 Vitamins/iron | | | | | | | | | | | |
| 1. Mention 5 commonly used drugs in each of the classes below (Sold or prescribed by you) | | | | | | | | | | | | |
| 🞏1 Vaccines | 🞏a…………… 🞏b………….. 🞏c…………..🞏d………….🞏e………………. | | | | | | | | | | | |
| 🞏2 Anthelmintic (Dewormers) | 🞏a…………… 🞏b………….. 🞏c…………..🞏d………….🞏e………………. | | | | | | | | | | | |
| 🞏3 Arachnidicides (ectoparasites) | 🞏a…………… 🞏b………….. 🞏c…………..🞏d………….🞏e………………. | | | | | | | | | | | |
| 🞏4 Antibiotics | 🞏a…………… 🞏b………….. 🞏c…………..🞏d………….🞏e………………. | | | | | | | | | | | |
| 🞏5 Vitamins/iron | 🞏a…………… 🞏b………….. 🞏c…………..🞏d………….🞏e………………. | | | | | | | | | | | |
| 1. Which months of the year do you achieve **most** sales/profit/ most cases (max 3) | 🞏1 Throughout the year  🞏2 Seasonal (Use calendar below) | | | | | | | | | | | |
|  | J | F | M | A | M | J | J | A | S | O | N | D |
|  |  |  |  |  |  |  |  |  |  |  |  |  |
| 1. Why to Q19 | ……………………………………………………………………………………………. | | | | | | | | | | | |
| 1. How do you sell/administer drugs to farmers | 🞏1 On prescription only  🞏2 Without prescription  🞏3 Both | | | | | | | | | | | |
| 1. For the case of **antibiotics**, and if **without prescription**, who decides on which antibiotic to give to the male farmer | 🞏1 Farmer  🞏2 Myself  🞏3 Extension agent of the area  🞏4 Other | | | | | | | | | | | |
| 1. For the case of **antibiotics**, and if **without prescription**, who decides on which antibiotic to give to the female farmer | 🞏1 Farmer  🞏2 Myself  🞏3 Extension agent of the area  🞏4 Other | | | | | | | | | | | |
| 1. What is the basis for deciding what antibiotic to give to your client/farmers (follow up of q21) | 🞏1 Symptoms as explained by the farmer and seen/verified by me  🞏2 Laboratory test results provide by the farmers / Done by me  🞏3 Advice given to the farmer by his animal health service provider  🞏4 Own judgment following farmers explanation  🞏5 Other (specify) | | | | | | | | | | | |
| 1. How is the dosage determined when advising the farmer? | 🞏1 As indicated on the drug  🞏2 My own judgment based on experience of success  🞏3 Estimated weight of the animal by farmer/ You  🞏4 Other(specify) | | | | | | | | | | | |
| 1. Most frequent way of selling the drug?   **(drug stockist)** | 🞏1 Single tablets / measurement depending on the farmer’s capacity to purchase  🞏2 Whole package for whole course of treatment  🞏3 Other (specify) | | | | | | | | | | | |
| 1. Most frequent way of administering the drug?   **(practitioner)** | 🞏1 Single dose / one-time measurement depending on the farmer’s capacity to pay  🞏2 Whole course of treatment as recommended **+** follow-up  🞏3 Other (specify) | | | | | | | | | | | |
| 1. Have your customers ever complained about drug failure? | 🞏 Yes 1 🞏 No 2 | | | | | | | | | | | |
| 1. If yes, for which drug? And how often? | 🞏1 Name drug 1………………. 🞏a)One 🞏b)twice 🞏c)three time 🞏d)more than three times  🞏2 Name drug 2………………. 🞏a)One 🞏b)twice 🞏c)three time 🞏d)more than three times  🞏3 Name Drug 3………………..🞏a)One 🞏b)twice 🞏c)three time 🞏d)more than three times  🞏4 Name drug 4………………… 🞏a)One 🞏b)twice 🞏c)three time 🞏d)more than three times | | | | | | | | | | | |
| 1. What do you do with expired drugs? | 🞏1 Discard  🞏2 Never experienced  🞏3 Return to National Dru Authority  🞏4 Return to the whole seller  🞏5 Sell to clients at cheaper cost | | | | | | | | | | | |

**Section C: Knowledge Attitude and Practices about AMR**

| 1. Have you ever heard about the antibiotic resistance phenomenon? | 🞏 Yes 1 🞏 No 2 |
| --- | --- |
| 1. If yes, from which channel? | 🞏1 Learned about AMR from my background training  🞏2 Heard from radio  🞏3 Learnt from a colleague  🞏4 Learnt from a short training/workshop  🞏5 Red from newspaper  🞏6 Learnt from television  🞏7 Learnt from reading in internet  🞏8 Other (specify) |
| 1. It is bacteria that can become resistant to antibiotics | 🞏1 Agree 🞏 2 Disagree 🞏3Don’t know |
| 1. It is people who can become resistant to antibiotics | 🞏1 Agree 🞏 2 Disagree 🞏3Don’t know |
| 1. Animals can become resistant to antibiotics | 🞏1 Agree 🞏 2 Disagree 🞏3Don’t know |
| 1. Antibiotic resistance is due to normal use of antibiotics | 🞏1 Agree 🞏 2 Disagree 🞏3Don’t know |
| 1. Antibiotic resistance is due to using antibiotics when they are not indicated | 🞏1 Agree 🞏 2 Disagree 🞏3Don’t know |
| 1. Antibiotics are effective in treating bacterial infections (e.g. Tuberculosis) | 🞏1 Agree 🞏 2 Disagree 🞏3Don’t know |
| 1. Antibiotics are effective in treating viral infections (e.g. New castle disease). | 🞏1 Agree 🞏 2 Disagree 🞏3Don’t know |
| 1. Antibiotics are effective in treating protozoal infections (e.g. ECF) | 🞏1 Agree 🞏 2 Disagree 🞏3Don’t know |
| 1. Antibiotics are effective in treating parasites infections (ex. Worms) | 🞏1 Agree 🞏 2 Disagree 🞏3Don’t know |
| 1. Antibiotics are effective in treating pain and inflammation | 🞏1 Agree 🞏 2 Disagree 🞏3Don’t know |
| 1. Antibiotics are effective in boosting animal growth | 🞏1 Agree 🞏 2 Disagree 🞏3Don’t know |
| 1. Antibiotics residues from animals can be found in meat | 🞏1 Agree 🞏 2 Disagree 🞏3Don’t know |
| 1. Antibiotics residues from animals can be found in milk | 🞏1 Agree 🞏 2 Disagree 🞏3Don’t know |
| 1. Antibiotics residues from animals can be found in eggs | 🞏1 Agree 🞏 2 Disagree 🞏3Don’t know |
| For **questions (49-53),** Resistant bacteria from animals can be transferred to humans through; | |
| 1. Consuming animal products (meat/milk/egg) of treated animal with antibiotics | 🞏1True 🞏2False 🞏3Don’t know |
| 1. Direct contact with the body of the animal | 🞏1True 🞏2False 🞏3Don’t know |
| 1. Direct contact with the faeces of animal | 🞏1True 🞏2False 🞏3Don’t know |
| 1. Direct contact with the body fluid of animals (saliva, blood) | 🞏1True 🞏2False 🞏3Don’t know |
| 1. Through the air | 🞏1True 🞏2False 🞏3Don’t know |
| 1. Do you know about drug’s withdrawal periods? | 🞏1 Yes 🞏2 No |
| 1. If yes to (q54), observation of drug withdrawal makes the animal products safer for consumption (meat, milk and eggs) | 🞏1 Agree 🞏 2 Disagree 🞏3Don’t know |
| 1. If yes to (q54), observation of drug withdrawal makes the animal recover from disease faster | 🞏1 Agree 🞏 2 Disagree 🞏3Don’t know |

**Section D: Policy and knowledge gaps**

| 1. I am conversant with the veterinary drug policy document of my country | 🞏1 Agree 🞏 2 Disagree 🞏3Don’t know |
| --- | --- |
| 1. The control of AMR in livestock is the role of the Government ONLY | 🞏1 Agree 🞏 2 Disagree 🞏3Don’t know |
| 1. The control of AMR in livestock is the role of both the Government AND all actors of the livestock value chain including myself. | 🞏1 Agree 🞏 2 Disagree 🞏3Don’t know |
| 1. The control of AMR in livestock is the role of actors of the livestock farmers ONLY | 🞏1 Agree 🞏 2 Disagree 🞏3Don’t know |
| 1. What is your most urgent need in relation to AMR? (single choice) | 🞏1 Knowledge on how to use antibiotics  🞏2 Understand mechanism of antibiotics resistance  🞏3 Knowledge on when to prescription of antibiotics  🞏4 understand links between the health of humans  animals and the environments  🞏5 Understand the policies about the use of veterinary drugs in the country |
| 1. Which among the following actions you think is critical for the sustainable control of AMR in livestock systems in Uganda? | 🞏1 More strong and directed policies on antibiotic use  🞏2 Raise awareness of farmers about the impact of misuse of antibiotics  🞏3 Strict monitoring of drug import in the country  🞏4 Re-enforce disease control in livestock  🞏5 Enhance disease diagnostic in livestock  🞏6 Strengthen quality control of drug stockists in the country |

Thank the respondent for his/her time and kind cooperation.

Did the respondent cooperate? 🞏Yes 🞏 No

Was the interview interrupted? 🞏Yes 🞏No
